# Research progress and hot spot analysis related to oxidative stress and osteoarthritis: a bibliometric analysis

**DOI:** 10.1186/s12891-023-06324-x

**Published:** 2023-05-23

**Authors:** Jin-Yu Gu, Fei Han, Si-Yu Chen, Qing Zhang

**Affiliations:** 1 Department of Orthopaedic, The Hospital of Wang Jing, China Academy of Chinese Medical Sciences, Huajiadi Street, Chao Yang District, Beijing, 100102 China; 2 Department of Orthopaedic, The Hospital of Guang An Men, China Academy of Chinese Medical Sciences, Beixian Ge Street，Xicheng District, Beijing, 100053 China

**Keywords:** Oxidative stress, Osteoarthritis, Bibliometric analysis, biclustering analysis, Hotspots

## Abstract

**Background:**

Osteoarthritis, a common degenerative osteochondral disease, has a close relationship between its mechanism of occurrence and oxidative stress. However, there are relatively few relevant studies in this field, and a more mature research system has not yet been formed.

**Methods:**

By searching the Web of Science (WOS) database, we obtained 1 412 publications in the field of osteoarthritis and oxidative stress. The search results were then analyzed bibliometrically using Citespace and VOSviewer, including a study of publication trends in the field, analysis of core authors, analysis of countries and institutions with high contributions, analysis of core journals, and to identify research trends and hot spots in the field, we performed keyword clustering.

**Results:**

We collected 1 412 publications on the field of osteoarthritis and oxidative stress from 1998–2022. By analyzing the publication trends in the field, we noted an exponential increase in the number of publications per year since 2014. We then identified the core authors in the field (Blanco, Francisco J., Loeser, Richard F., Vaamonde-garcia, et.al) as well as the countries (China, USA, Italy et.al) and institutions (Xi An Jiao Tong Univ, Wenzhou Med Univ, Zhejiang Univ et.al). The OSTEOARTHRITIS AND CARTILAGE and INTERNATIONAL JOURNAL OF MOLECULAR SCIENCES contain a large number of research papers in this field, and through keyword co-occurrence analysis, we counted 3 227 keywords appearing in the field of osteoarthritis and oxidative stress. These keywords were clustered into 9 groups, representing 9 different research hotspots.

**Conclusions:**

Research in the field of osteoarthritis and oxidative stress has been developing since 1998 and is now maturing, but there is an urgent need to strengthen international academic exchanges and discuss the future focus of research development in the field of osteoarthritis and oxidative stress.

**Supplementary Information:**

The online version contains supplementary material available at 10.1186/s12891-023-06324-x.

## Introduction

Osteoarthritis is a common degenerative joint disease that occurs mainly in people over 50 years of age and is more common in women. Its onset is closely related to increasing age [1]. After the onset of osteoarthritis, as the disease progresses, the joint surfaces become poorly anastomosed, pain occurs during weight bearing and rest, and muscle spasm and atrophy trigger functional impairment in patients [2, 3]. Once osteoarthritis is not controlled effectively and in a timely manner, it can lead to scoliosis and curvature of the thoracic spine [4], and deformation of several parts of the body, including the joints and cervical spine. In severe cases, it can affect heart function, intestinal function, and lead to cardiovascular, cerebrovascular and hypertensive diseases [5–7]. The quality of life and prognosis of patients are seriously affected.

Oxidative Stress is a state in which there is an imbalance between oxidative and antioxidant actions in the body, tending to oxidation, leading to inflammatory infiltration of neutrophils, increased secretion of proteases, and production of large amounts of oxidative intermediates, which is an important factor in aging and disease [8, 9]. Oxidative stress due to excess reactive oxygen species is closely associated with the development of bone-related diseases such as osteoarthritis, osteoporosis, intervertebral disc degeneration, rheumatoid arthritis and osteosarcoma, and oxidative stress is a key pathogenesis of bone-related diseases [10]. Basic biological research has revealed that free radicals are harmful compounds produced during oxidation reactions in the human body, the most common of which are superoxide anion radicals, which are strongly oxidizing and lead to increased cellular fragility and impairment of the original cellular function by altering the fluidity of the membrane body, reducing cellular deformability and increasing membrane permeability [11, 12]. Articular cartilage is mainly composed of chondrocytes that maintain function and a matrix that contains water, proteoglycans and collagen [13, 14]. The damaging effect of free radicals is more evident in the damage of human articular cartilage. By comparing changes in inflammatory factors such as ROS in healthy people and patients with osteoarthritis, some scholars have found that oxygen free radicals play an important role in the pathological progression of osteoarthritis [15–17].

Oxidative stress also has an important role in the progression of age-induced osteoarthritis. As we age, chondrocytes undergo progressive senescence, leading to decreased responses to growth factors, mechanical stimuli, etc., increasing the risk of osteoarthritis [18, 19]. It has been found that oxidative stress can contribute to chondrocyte senescence by shortening telomerase. Oxidation of cartilage collagen leads to collagen cleavage, which changes the original properties of collagen fibers and increases fiber fragility, making the body susceptible to fatigue damage and thus increasing the incidence of osteoarthritis [20, 21]. Mitochondrial damage is also an important cause of osteoarthritis, and oxidative stress plays an integral role in this. On the one hand, oxidative stress generates free radicals that directly attack mitochondria, and on the other hand, oxidative stress-induced cellular senescence exacerbates mitochondrial metamorphosis [21]. Mitochondria are the core of energy metabolism, so damage to mitochondria directly affects energy and DNA and RNA synthesis, leading to the arrest of cell development and growth, which in turn leads to the development of osteoarthritis [22, 23]. The high prevalence and disability of osteoarthritis seriously affects human health, and oxidative stress, as an important pathogenesis, has received much attention from international experts. In the past, academic research in this field has focused on the pathogenesis, influencing factors and treatment, but due to the complexity of the disease, the focus of future research is likely to remain on these aspects.

Bibliometrics is a discipline that uses mathematics, statistics and other measurement methods to study the distribution structure, quantitative relationships, laws of change and quantitative management of literature and intelligence, and thus explore certain structures, characteristics and laws of science and technology [24]. Vosviewer and Citespace is a citation visualization software developed in the context of bibliometrics and data visualization to present the structure, patterns and distribution of scientific knowledge through visualization. Within the last two decades, no scholar has analyzed the field of osteoarthritis and oxidative stress using a bibliometric approach, and we are the first to perform a systematic bibliometric analysis of the research content in this field.

The purpose of our work is to analyze the Web of Science database for research related to osteoarthritis and oxidative stress through VOSviewer and Citespace, to analyze the scientific output and its evolution in the field of arthritis and oxidative stress worldwide and in major countries or regions and among core scholars, and to find influential journals in the field as well as past research hotspots and relevant topics. To achieve our objectives, we will conduct the following analyses: (1) describe the publication trends in the field of osteoarthritis and oxidative stress between 1998 and 2022 (2) analyze the research contributions of different countries in the field (3) analyze the research contributions of different institutions in the field (4) search for core research scholars in the field and describe their research directions (5) search for influential journals in the field journals in the field (6) cluster analysis of popular research directions in the field (7) analysis of the evolution of different research hotspots.

With Vosviewer, we present a summary of research in the field of osteoarthritis and oxidative stress from 1998–2022, analyzing trends in the field by analyzing the contributions made by different countries, journals, institutions, and scholars in the field.

## Materials and methods

### Data sources and search strategy

We searched the Web of Science (WoS) database using the keywords oxidative stress, antioxidative stress, osteoarthritis, and degenerative Arthritis. The specific search strategy was: ("Oxidative Stress" [Topic] OR ("antioxidative stress" [Topic]) AND ( "Osteoarthritis" [Topic] OR "Degenerative Arthritis" [Topic]). Publication dates were restricted from January 1998 to September 2022. The search strategy was repeated twice by two researchers independently and cross-checked to prevent search bias and omissions.

### Inclusion criteria

**Fig. 1:**
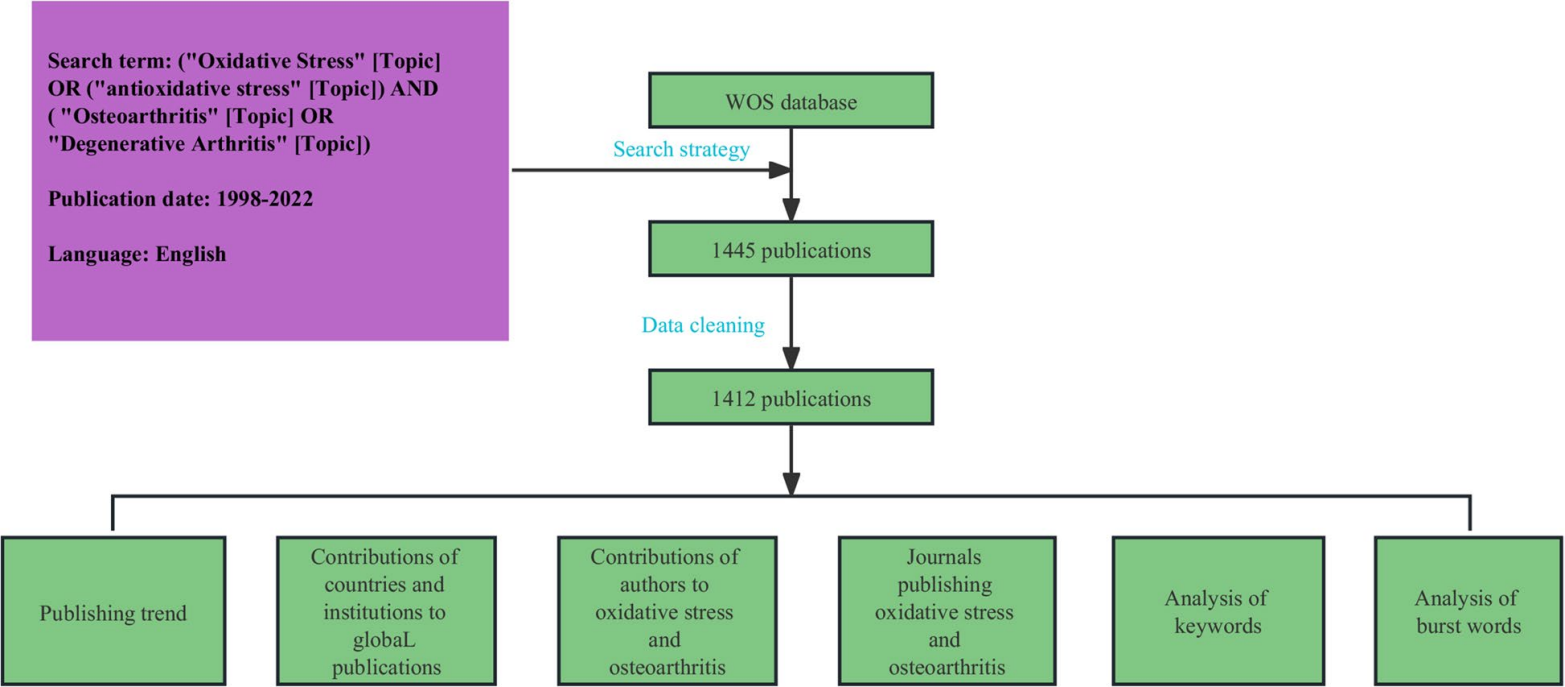
Data processing flow chart of bibliometric analysis

All documents used for bibliometric analysis met the following criteria: (1) The documents were published between January 1998 and December 2021. (2) The language of publication was English. (3) The article type is “Article” or “Review Article” The literature search and selection process is described in Fig. 1.

### Data collection

The title, author, institution, year of publication, country, and MeSH term/MeSH subheading were included. By searching the database, we obtained 1 445 relevant papers, which were then screened according to the inclusion and exclusion criteria mentioned above, and 1 412 papers were finally included in the analysis. These documents were saved as "full record and citation references" and "plain text", to generate the source files for analysis.

### Data processing software

Microsoft Excel (software version 2018, Microsoft Corporation, Redmond, WA, USA) allows pre-processing of data, which includes programming errors, incorrect recording and correction or elimination of files that are inconsistent with the subject matter. In addition, we used the software to plot statistical graphs of paper publication trends and tables of information on authors, subject areas, and number and quality of papers [25].

VOSviewer (software version 1.6.18, Leiden University's Centre for Science and Technology Studies (CWTS), Amsterdam, The Netherlands) VOSviewer (software version 1.6.18, Leiden University's Centre for Science and Technology Studies (CWTS), Amsterdam, The Netherlands) is one of the many scientific knowledge mapping software, i.e., the mapping of scientific knowledge through the relationship construction and visual analysis of "network data" (mainly literature knowledge units), showing the structure, evolution, cooperation and other relationships of knowledge domains. The core idea of VOSviewer software design is "co-occurrence clustering", that is, two things appearing at the same time means they are related to each other; there are many types of such related relationships, and their strength and direction are different; based on the clustering of relationship strength and direction measures, different types of groups can be found. We use this software to perform country, institution, author, journal and keyword clustering analysis [26, 27].

Citespace (software version 5.6 R5, Drexel University, Philadelphia,PA, USA) is a web-based Java application for data analysis and visualization. It is a unique and influential application in the field of information visualization and analysis. The Citespace software includes co-citations, co-authors and co-occurring keywords, which help provide direction for analyzing a research area. It is mainly used in our research for burst words analysis [28].

### Statistical analysis

A literature reading database was created through NoteExpress 3.0 software, and the results of the above search-based detection of literature titles were imported into the database and screened and organized according to inclusion criteria. The database was created using Excel 2018 software to perform a bibliometric analysis of the literature in the field of oxidative stress and osteoarthritis in terms of volume of articles, authors, journals, countries, institutions, and others from 1998–2022. and clustering analysis of keywords using VOSviewer and Citespace software to provide a reference for researchers in the field of osteoarthritis to grasp the hot spots and frontiers of research in the field. In addition, this study mainly used numbers and percentages to statistically describe the data, so analysis of difference was not used.

## Results

### Publishing trend

**Fig. 2:**
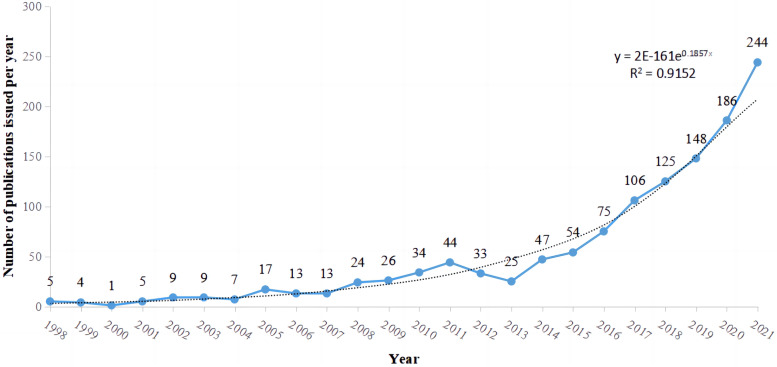
Visual graph of publication trend

After searching the WOS database and excluding irrelevant literature, a total of 1 412 publications were included in our study. Using Excel to record the number of publications per year and create a statistical chart to analyze the number of publications in this field per year, we found that publications in the field of osteoarthritis and oxidative stress generally showed an increasing trend from 1998 to 2021, with only small fluctuations in the number of publications in some years (2000, 2005) and a relatively smooth growth trend. However, from 2012 to 2013, the number of publications in this field declined significantly, probably due to a bottleneck in the field of osteoarthritis and oxidative stress, where no breakthrough findings have been found. Subsequently, the number of publications increased significantly from 2014 onwards (see Fig. 2).

The first study published in this field was on the effect of redox status on rheumatoid arthritis published in 1997 [29]. This study found that altered redox status had an important role in the hyporesponsiveness of joint T cells in patients with rheumatoid arthritis, revealing part of the mechanism by which oxidative stress causes rheumatoid arthritis. Subsequently, research in this area became widespread. Starting in the 2000s, researchers in various countries began to explore the relationship between oxidative stress and the development of osteoarthritis, with only a few dozen annual publications at first, focusing on finding evidence of an altered oxidative state in patients with osteoarthritis [30–33]. Then starting in 2010, research focused on the mechanistic role of osteoarthritis and oxidative stress, looking for its associated molecular mechanisms, and the number of annual publications increased substantially, with many researchers becoming involved [34, 35]. From 2015 to the present, research in this field has begun to focus on the study of different drugs for improving the progression of the disease in patients, exploring the mechanisms of action of some drugs [36–39]. Osteoarthritis has attracted a great deal of attention due to its harmful effects on the human body and its serious impact on people's healthy lives. The current research theme in this field is still the study of the mechanism of osteoarthritis and oxidative stress and the search for different drugs to slow down the progression of osteoarthritis by inhibiting oxidative stress, and it is believed that the research in this field will continue to develop in the future.

In order to understand the growth pattern of literature related to osteoarthritis and oxidative stress at home and abroad more accurately and scientifically, we applied the bibliometric theory and WoS database as the basis to conduct a statistical study on the growth pattern of literature related to osteoarthritis and oxidative stress at home and abroad in a time series. In order to assess whether scientific production in the field of osteoarthritis and oxidative stress complies with the law of exponential growth, we tested it with a regression model and obtained the model that is best adjusted to the data. According to Price's law, since the value of R^2^ (coefficient of determination) is 0.915. the value of this coefficient close to 1 guarantees that the number of publications in this research area is growing.

### Contributions of countries to global publications.

**Fig. 3:**
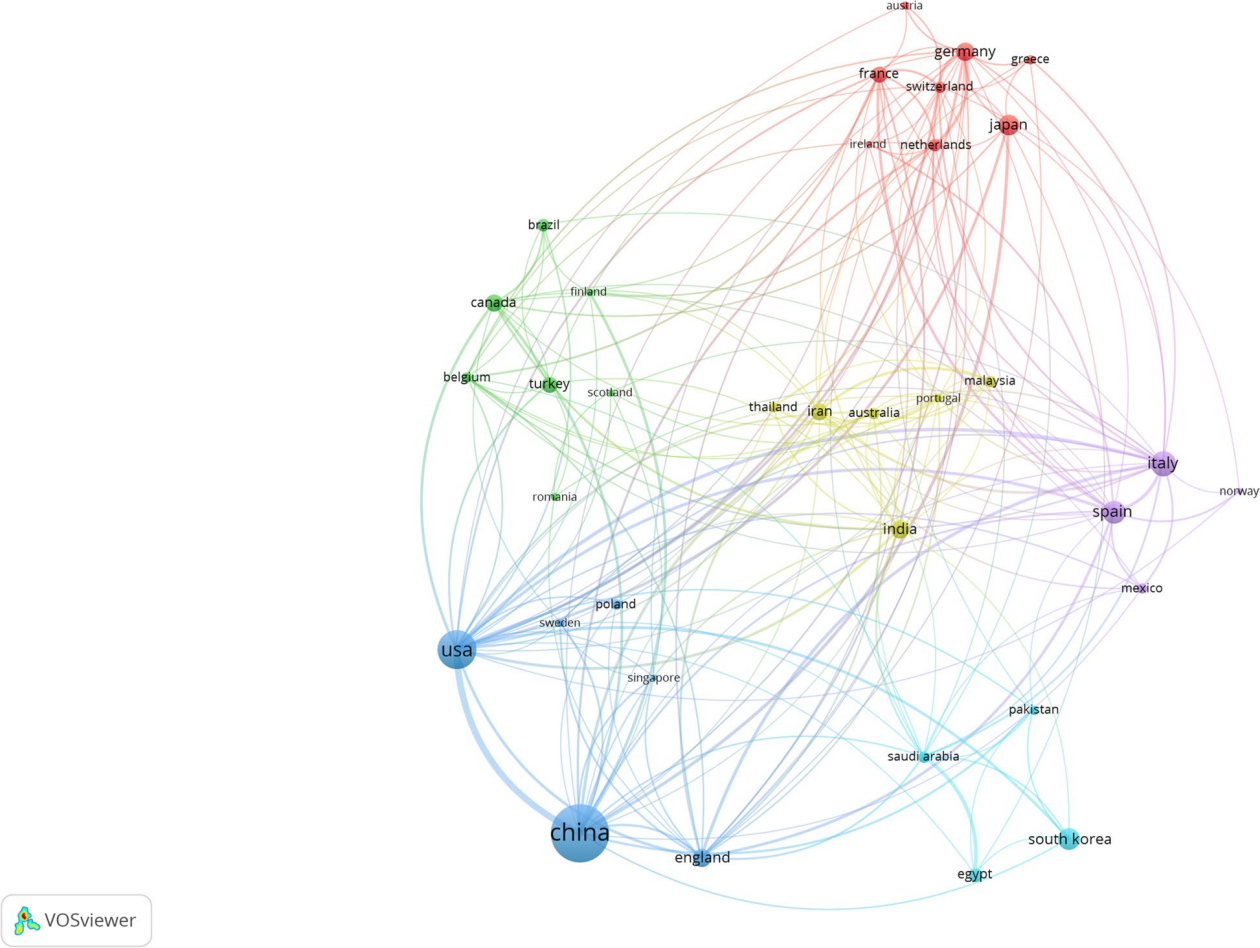
Country Visualization Chart

The analysis revealed that 74 countries published articles in the field of oxidative stress and osteoarthritis in 1998–2022. Table 1 shows the top 10 countries that published articles in the field of oxidative stress and osteoarthritis. In order to better observe the number of publications between different countries and the collaboration between different countries in this academic field, we used the Co-authorship-Countries function of VOSviewer to analyze and visualize (see Fig. 3), and we set the number of publications in a country to be presented in our results when it is greater than or equal to 5. 36 countries published more than 5 papers in the field of oxidative stress and osteoarthritis in 1998–2022. From Table 1 and Fig. 3, we find that China has published papers with Canada, Spain, Italy, the United States, the United Kingdom, Germany, Japan, India, South Korea, the Netherlands, the United Kingdom, Iran, Thailand, New Zealand and other countries/regions, and has common publications with these countries. The main research contents of the United States, Italy and China are respectively interleukin 1, low back pain, and nf-kappa b. These three countries cooperate closely, so it can be inferred that inflammation and immunity are the common research themes of these three countries. Developed countries are able to solve these problems based on their strong economic and technological strength.

**Table 1:** The top 10 countries contributing to publications

Rank	Country	Articles	Total number of citations	Average number of citations
1	China	511	7648	15.0
2	Usa	228	10,878	47.7
3	Italy	98	2513	25.6
4	Spain	78	2806	36.0
5	South Korea	74	1702	23.0
6	Japan	63	2251	35.7
7	India	51	1496	29.3
8	Germany	50	2106	42.1
9	England	49	1822	37.2
10	Iran	47	1868	39.7

### Contributions of institutions to global publications

**Fig. 4:**
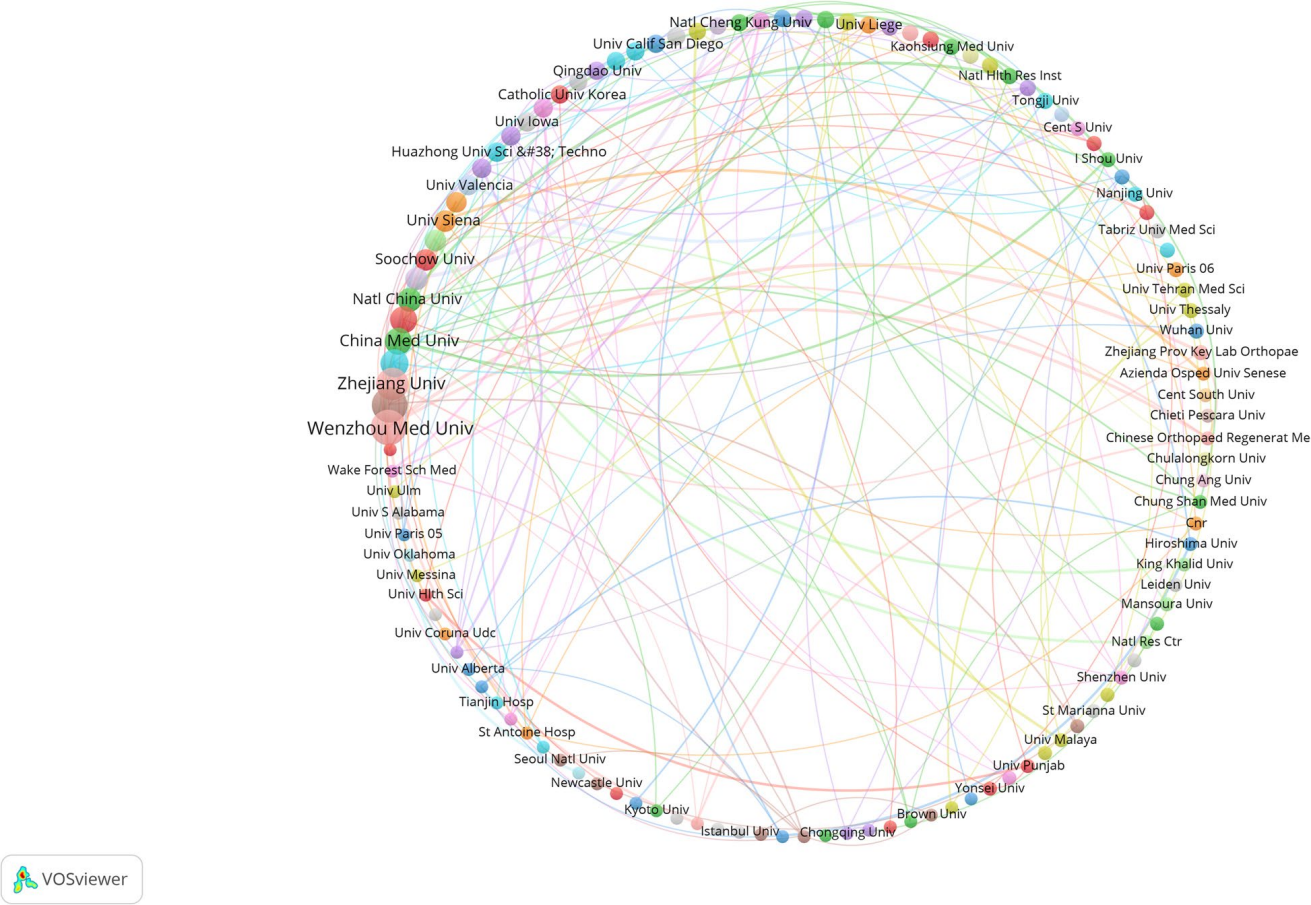
Visualization of institutional posting volume

The main research institutions in the field of oxidative stress and osteoarthritis were analyzed and visualized using VOSviewer. A total of 1 823 institutions published research papers in this field, with 119 institutions publishing more than 5 papers. Table 2 shows the top ten contributing institutions, along with the total number of citations and the average number of citations of their publications, ranked by the time of the most recent publication if they have the same number of publications. Figure 4 shows the visualization of contribution and collaboration of different institutions. Cooperation networks have been formed among different institutions with Xi 'an Jiaotong University, Zhejiang University and Wenzhou Medical University, China Medical University and National University of Defense Technology, and etc. as the cores. The major research interests of most institutions fall into six broad categories: mitophagy, kashin-back disease, pkr, mental health, ginger, and galactosidase. Institutions that work closely together have common research topics.

**Table 2:** The top 10 institutions contributing to publications

Rank	Institution	Articles	Total number of citations	Average number of citations
1	Xi An Jiao Tong Univ	36	510	14.2
2	Wenzhou Med Univ	36	701	19.5
3	Zhejiang Univ	30	502	16.7
4	Shanghai Jiao Tong Univ	23	447	19.4
5	Nanjing Med Univ	20	264	13.2
6	China Med Univ	20	204	10.2
7	Natl China Univ	16	237	14.8
8	Univ Bologna	15	424	28.3
9	Soochow Univ	14	91	6.5
10	Univ Siena	13	192	14.8

### Contributions of authors to oxidative stress and osteoarthritis

**Fig. 5:**
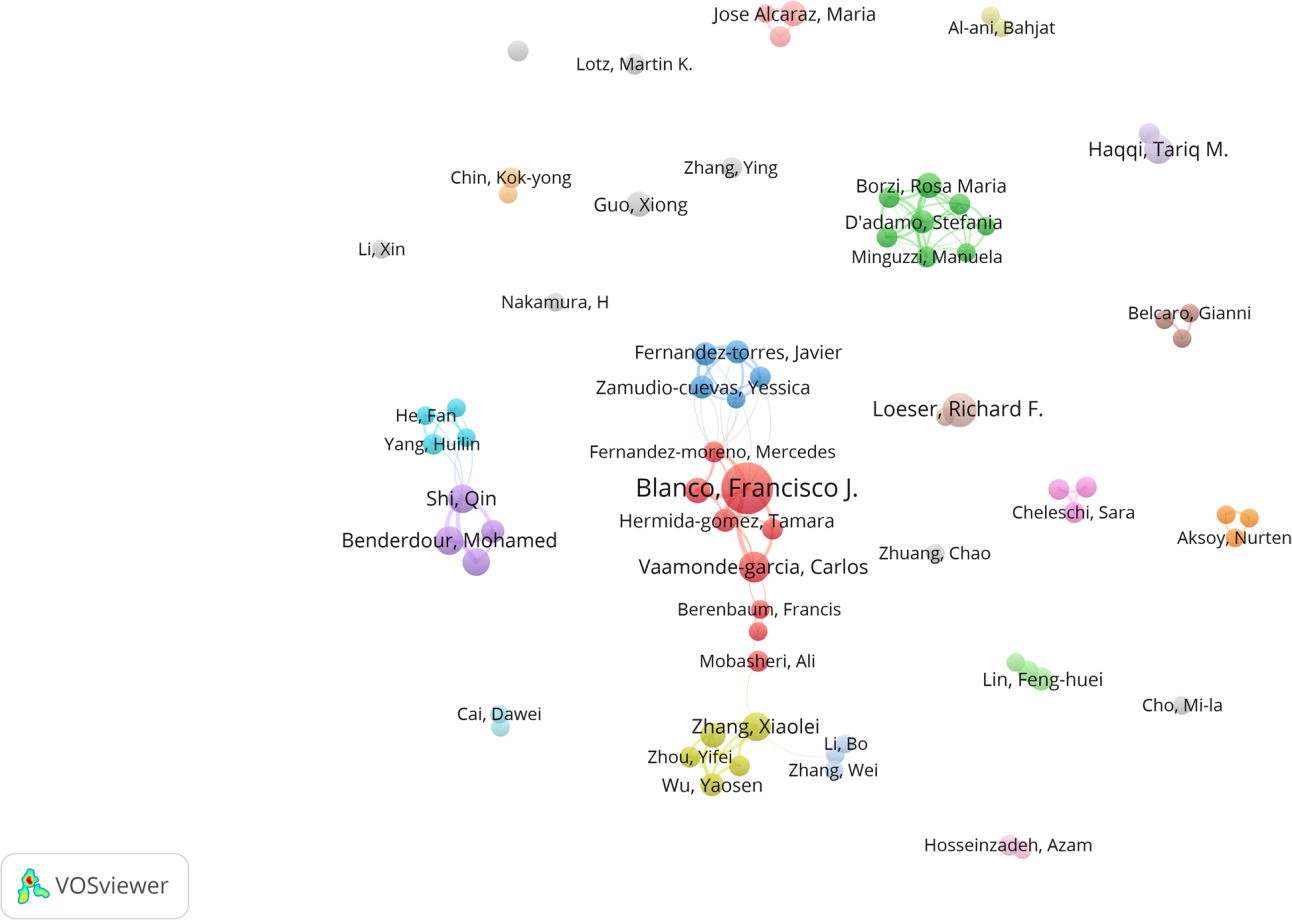
Authors visualization map

To identify representative scholars and core research strengths in the field of osteoarthritis and oxidative stress, we performed an authorship analysis using the co-authorship function of VOSviewer. Based on data from the WOS database from 1998–2022, a total of 7 507 authors published research papers on the field during this period. With such a large group of authors, it is necessary to identify the core authors in the field, who are the backbone of a research field and whose research results often represent the major research results in the field. According to Lotka's law: *the number of papers published by the least prolific of the core authors is equal to 0.749 times the square root of the number of papers published by the most prolific scientists* [40]. Therefore, authors with 5 or more publications in the field of osteoarthritis and oxidative stress were defined as core authors. There were 73 core authors with a total of 477 publications, accounting for 33.8% of the total publications, and a stable group of authors has not yet been formed in this field. Table 3 shows the top 10 most productive authors in the field in terms of number of publications and the number of citations of articles published by these scholars. Collaborations often exist between core authors. Figure 5 shows a visualization of core authors and collaborations in the field of osteoarthritis and oxidative stress. The nodes in the graph represent scholars with more than or equal to 5 publications, and the number of nodes is 73, meaning that there are 73 core authors. The size of the nodes represents the number of publications, the larger the node, the more the scholar has published in this field, and the connections between the nodes represent the degree and pattern of collaboration between different scholars. The clusters formed by different colors in the visualization graph represent the research focused on the same direction in the field among different scholars in that cluster.

**Table 3:** The top 10 authors contributing to publications

Rank	Author	Articles	Total number of citations	h-index	Affiliation
1	Blanco, Francisco J	26	949	62	Spain
2	Loeser, Richard F	13	1731	61	USA
3	Vaamonde-garcia, Carlos	11	255	12	Spain
4	Benderdour, Mohamed	10	286	32	Canada
5	Haqqi, Tariq M	10	600	47	USA
6	Shi, Qin	10	268	20	China
7	Zhang, Xiaolei	10	214	32	China
8	Fahmi, Hassan	9	274	39	Canada
9	Borzi, Rosa Maria	8	223	27	Italy
10	Guo, Xiong	8	166	19	China

Among the core authors, the one with the most publications is Blanco Francisco J. From 1998 to 2021, this scholar has published 26 papers with 949 citations, with an average of 36.5 citations per paper, and Blanco Francisco J is also the scholar with the closest collaboration with other scholars. By analyzing his publications, we found that most of his research is on the pathogenesis of osteoarthritis, especially the role of mitochondrial DNA in osteoarthritis. For example, he proposed that genetic manipulation of mtDNA ameliorated joint aging damage in a homogenized mouse model, suggesting that mtDNA variability is a prognostic factor for aging-related osteoarthritis and that regulation of mitochondrial oxidative phosphorylation (OXPHOS) may be a novel therapeutic target for the treatment of aging-related osteoarthritis [41]. In second place is Loeser, Richard F., with 13 publications and 1731 citations, with an average of 133.2 citations per article. The most important theories or methodologies in the field of research. The main focus of this scholar's research is to investigate the basic research on osteoarthritis and the pathways involved in osteoarthritis due to aging of the body during the aging process [42, 43].

### Journals publishing oxidative stress and osteoarthritis

**Fig. 6:**
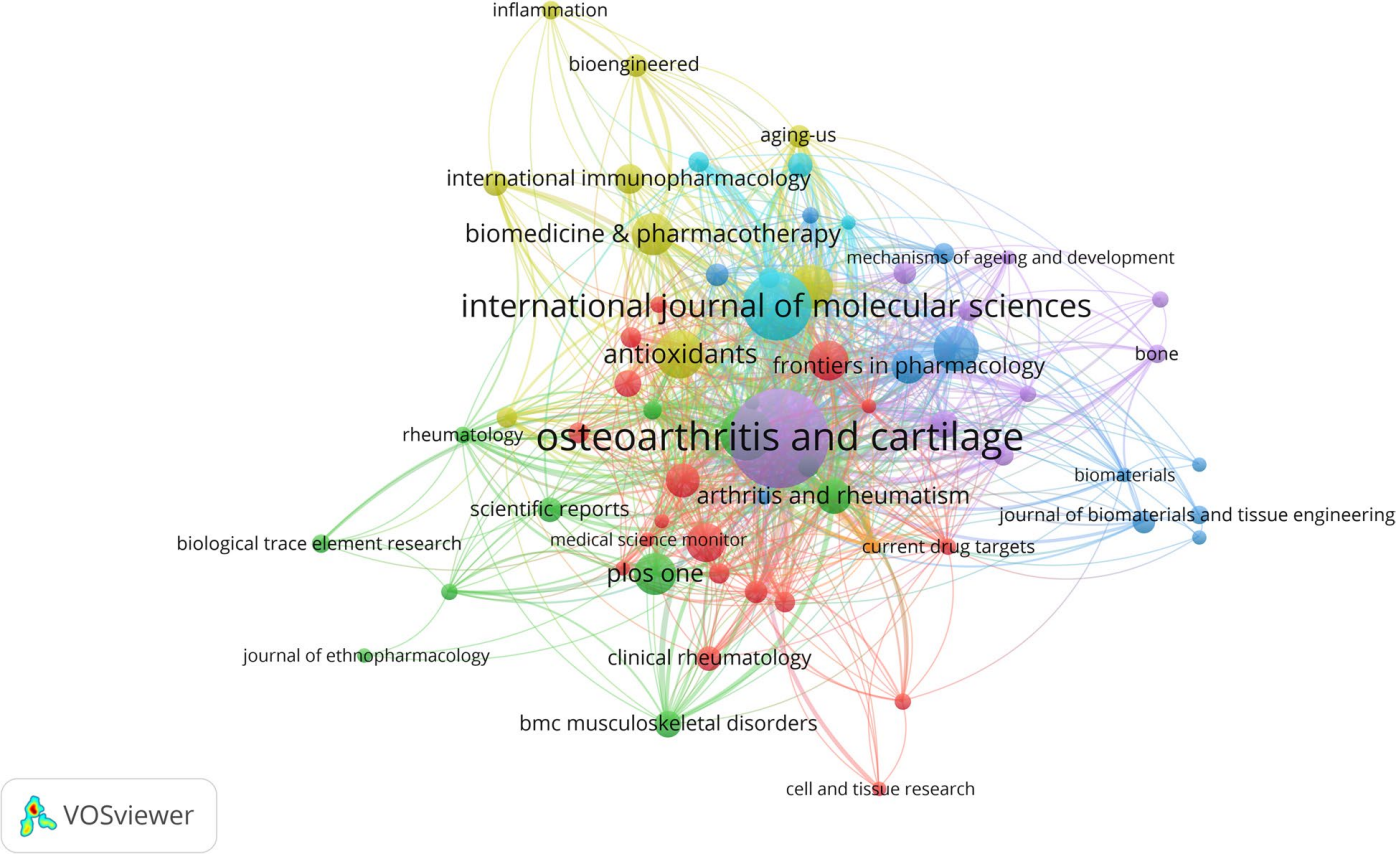
Visualization of journal article volume

Figure 6 visualizes the distribution of journals, with the size of the nodes representing the number of articles. We found that most of the journals that included papers in the field of oxidative stress and osteoarthritis belonged to the field of osteopathy and rheumatic diseases, except for a few comprehensive journals. Table 4 shows the top ten journals in terms of number of articles. The top three journals with the highest number of articles were osteoarthritis and cartilage, international journal of molecular, and arthritis research & therapy, and analyzing these results we know that most of the articles in the field of oxidative stress and osteoarthritis were published in these journals. We analyzed the average number of citations of articles published in the top 10 journals, and found that, except for osteoarthritis and cartilage and arthritis research & therapy, the other journals received more articles in this field, but the average number of citations was low, suggesting that the quality of literature received by these journals is relatively low. The quality of the literature received by these journals is relatively low. In view of this, Table 5 lists the top ten journals with the highest average number of citations to the literature, namely nature reviews rheumatology, arthritis and rheumatism and journal of biological chemistry. Most of the literature received by these three journals are high quality articles.

**Table 4:** The top 10 most cited journals with publications

Rank	Journal title	Publications (N)	Total number of citations	Average number of citations	IF
1	OSTEOARTHRITIS AND CARTILAGE	60	2982	49.7	7.5
2	INTERNATIONAL JOURNAL OF MOLECULAR SCIENCES	37	826	22.3	6.2
3	ARTHRITIS RESEARCH & THERAPY	26	1326	51	5.6
4	ANTIOXIDANTS	24	156	6.5	7.7
5	OXIDATIVE MEDICINE AND CELLULAR LONGEVITY	22	339	15.4	7.3
6	FREE RADICAL BIOLOGY AND MEDICINE	21	840	40	8.1
7	BIOMEDICINE & PHARMACOTHERAPY	20	728	36.4	7.4
8	PLOS ONE	20	424	21.2	3.8
9	MOLECULAR MEDICINE REPORTS	19	353	18.6	3.4
10	NUTRIENTS	18	370	20.6	6.7

**Table 5:** The top 10 most cited on average

**Rank**	**Journal title**	**Publications (N)**	**Total number of citations**	**Average number of citations**
1	NATURE REVIEWS RHEUMATOLOGY	8	2005	250.6
2	ARTHRITIS AND RHEUMATISM	16	1908	119.3
3	JOURNAL OF BIOLOGICAL CHEMISTRY	5	482	96.4
4	CURRENT OPINION IN RHEUMATOLOGY	6	533	88.8
5	BIOCHEMICAL PHARMACOLOGY	5	400	80.0
6	ANNALS OF THE RHEUMATIC DISEASES	7	498	71.1
7	JOURNAL OF PINEAL RESEARCH	5	300	60.0
8	RHEUMATOLOGY INTERNATIONAL	5	282	56.4
9	ARTHRITIS & RHEUMATOLOGY	12	619	51.6
10	ARTHRITIS RESEARCH & THERAPY	26	1326	51.0

### Analysis of keywords

**Fig. 7:**
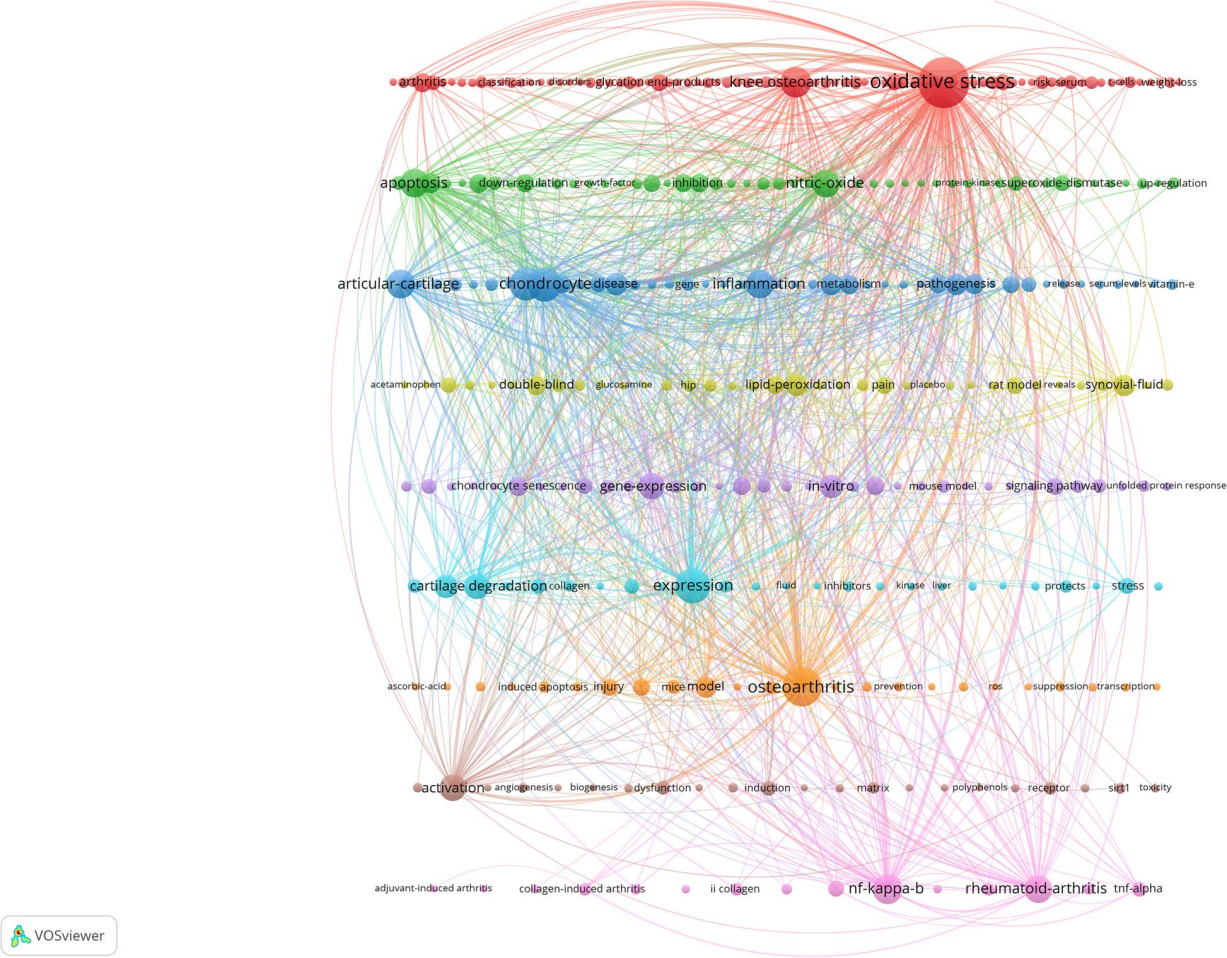
Keyword Clustering Map

In general, there must be some kind of correlation between several keywords given in a paper, and this association can be expressed by the frequency of co-occurrence. It is generally accepted that the more times a pair of words appears in the same document, the closer the two topics are. Keyword clustering uses the co-occurrence of word pairs or noun phrases in a collection to determine the relationship between topics in the discipline represented in the collection. Keywords represent the core knowledge of a paper, and keyword co-occurrence analysis can be used to discover the research hotspots in a scientific field. We used VOSviewer to draw a visual keyword co-occurrence network view of all the keywords involved in the retrieved literature. There are 3 227 keywords in total, and we set 332 keywords with the number of occurrences greater than or equal to 5 to be presented in the visualization graph, and the results are shown in Fig. 7. The circles in the figure are the the frequency of the keywords, and the higher the frequency of occurrence indicates that the research topic is more popular; the color of the nodes represents different clusters. In order to better understand the specifics of high-frequency keywords, Table 6 lists the top 20 keywords in terms of number of occurrences. As can be seen in Fig. 7 and Table 6, high-frequency keywords such as "oxidative stress", "osteoarthritis", "expression", "chondrocyte", "cartilage", "knee osteoarthritis", "nf-kappa-b", "inflammation", "apoptosis", and "articular-cartilage" constitute the representative terms in this field. Research hotspots were summarized using keyword biclustering technique analysis, and 9 clusters were generated from 332 high-frequency keywords, representing the 9 most popular research directions in the field of oxidative stress and osteoarthritis.

**Table 6:** The 20 most frequently occurring keywords

Rank	Keyword	Occurrences	Rank	Keyword	Occurrences
1	oxidative stress	636	11	rheumatoid-arthritis	140
2	osteoarthritis	318	12	nitric-oxide	139
3	expression	256	13	activation	128
4	chondrocyte	227	14	gene-expression	120
5	cartilage	206	15	cartilage degradation	118
6	knee osteoarthritis	180	16	in-vitro	94
7	nf-kappa-b	167	17	antioxidant	91
8	apoptosis	157	18	disease	81
9	inflammation	156	19	lipid-peroxidation	77
10	articular-cartilage	155	20	model	75

We analyzed these 9 clusters:

1. Cluster 1 focuses on the correlation between osteoarthritis, especially knee osteoarthritis, and oxidative stress and glycosylation end products.
2. Cluster 2 is about the regulatory mechanisms of nitric oxide and apoptosis in the development of osteoarthritis.
3. Cluster 3 is about the role played by factors such as articular cartilage, chondrocytes and inflammation in the pathology of osteoarthritis formation.
4. Cluster 4 is about double-blind experiments in animal models or humans to perform a bit of research related to osteoarthritis.
5. Cluster 5 is about improving cartilage degeneration by studying chondrocyte silencing of some signaling pathways to suppress related gene expression in an in vitro experiment.
6. Cluster 6 is about cartilage degradation in osteoarthritis and the expression of related collagen.
7. Cluster 7 is about how to prevent and suppress some of the damage and pathological changes associated with osteoarthritis.
8. Cluster 8 is about the role of activation of some proteins and biomodulators in the development of osteoarthritis
9. Cluster 9 is about the mechanism of NF-κB pathway in the development of rheumatoid arthritis, in addition, NF-κB pathway has a close relationship with other clusters directly.

### Analysis of burst words

**Fig. 8:**
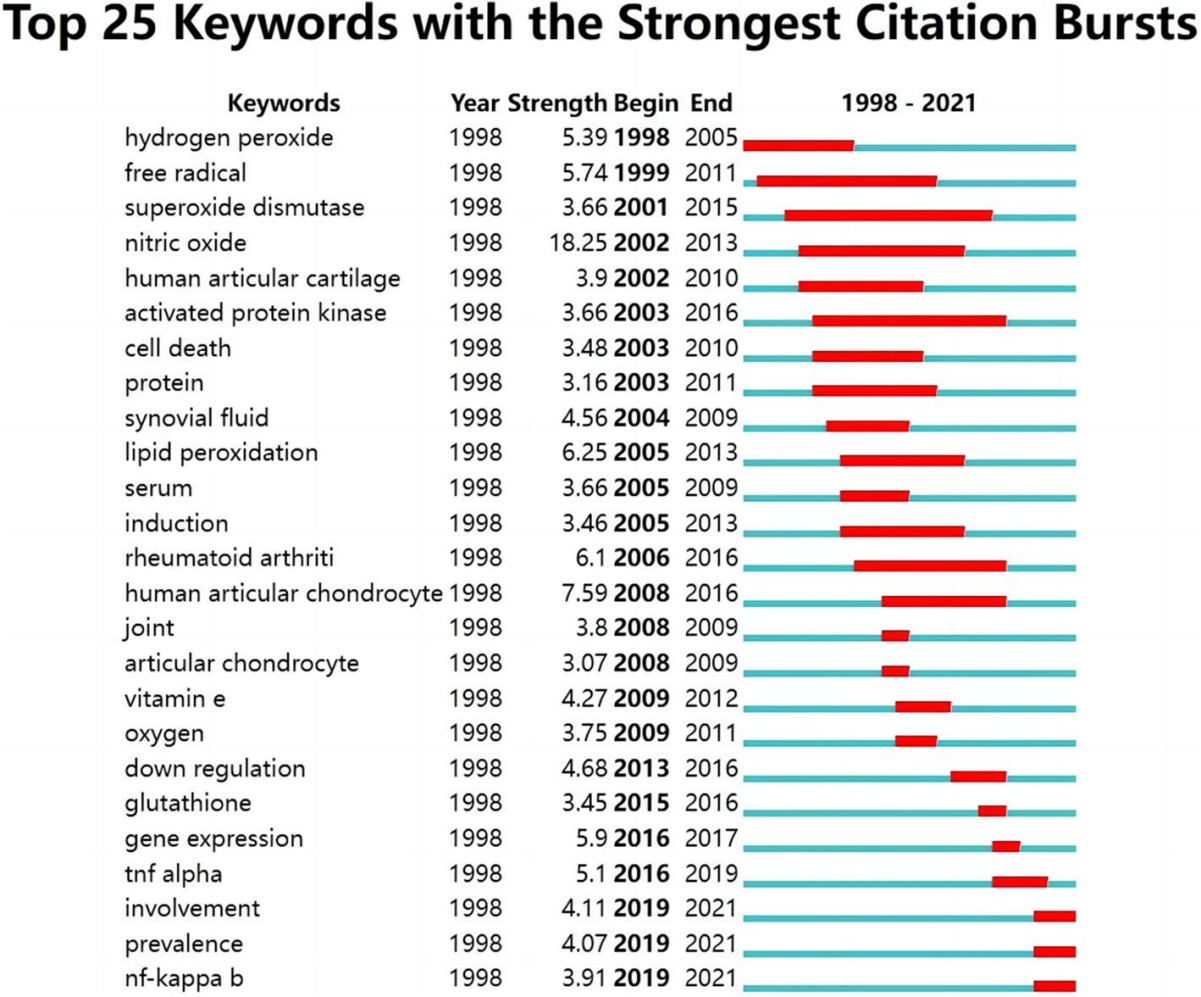
Burst words

We examined 1 412 records for burst words by collecting all literature related to the field of oxidative stress and osteoarthritis from 1998–2022. The burst of a keyword represents the frequent occurrence of that keyword in a certain period, and this information can not only suggest the change of research hotspots in that research area, but also can predict the future hotspot research by indicating the research trends in recent years. 25 burst keywords that were found are shown in Fig. 8. Hydrogen peroxide was the first to attract the interest of scholars, and It continued to receive attention until 2005. During this period, free radical, superoxide dismutase, etc. received attention one after another. Notably, the NF-κB pathway has been highlighted from 2019 until now, suggesting us that the direction of research between this pathway and osteoarthritis and oxidative stress may be a hot spot for future research.

## Discussion

From 1998–2022, the body of research in the field of oxidative stress and osteoarthritis slowly matured, with an exponential increase in the number of publications per year since 2016 and an increasing number of experts in orthopaedic science focusing their insights on this area. Although research in this area is extensive, the connections between research topics are more confusing. China has the most accurate number of publications, but the total frequency of citations in the United States is the most, indicating that the quality of published papers in this field in the United States is high, and China should strengthen the depth of research. Most of the top 10 institutions are from China, indicating that China has the most authoritative research institutions in this field. As can be seen from Fig. 5, there is less cooperation among different researchers, indicating that many researchers in this field may have less communication. Therefore, it is suggested to strengthen cooperation in future studies.

We used VOSviewer's biclustering technique analysis to analyze keywords into 9 clusters, and the analysis of these 9 clusters was used to sort out the research focus in the field of oxidative stress and osteoarthritis.

Cluster I focuses on the relationship between glycosylation end products and oxidative stress in osteoarthritis (especially osteoarthritis). Osteoarthritis, as a degenerative disease, increases in prevalence with age. Many scholars have focused their attention on the study of pathogenesis to find the causes of osteoarthritis due to ageing. As early as the beginning of research in this field, it was suggested that cellular and tissue changes due to the accumulation of Advanced glycation end products during aging may lead to diseases such as osteoarthritis [44]. In 2002 Drinda et al. first identified N(epsilon)-carboxymethyllysine (CML, a maker of oxidative stress) in the synovial tissue of patients with rheumatoid arthritis and osteoarthritis, demonstrating that CML plays an important role in rheumatoid arthritis and osteoarthritis and may be associated with triggering the autoimmune response [45]. Subsequently, research in this field began to search for treatments for advanced glycation end products for osteoarthritis and proposed the use of AGE inhibitors to slow down the progression of osteoarthritis and found that AGE inhibitors are used to attenuate glyco-oxidative stress through the basic mechanism of sequestration metal ions, reactive 1,2-dicarbonyl compounds, reactive oxygen species and reactive nitrogen species [46].

Cluster II is a discussion of the relationship between nitric oxide-related apoptosis and osteoarthritis. Previous studies have suggested that nitric oxide is a catabolic-related factor that mediates the expression of pro-inflammatory cytokines, inhibits collagen and proteoglycan synthesis and induces apoptosis to perpetuate the disease process in osteoarthritis [47]. In recent years, some scholars have conducted some experimental studies on nitric oxide to find drugs to inhibit the progression of osteoarthritis. Sun et al. found that trichothecene, a flavonoid, has antimetabolic, anti-inflammatory and anti-apoptotic effects in chondrocytes [48]. Chen et al. found that Juglanin has protective anti-inflammatory effects on human chondrocytes, and the mechanism may be the inhibition of IL-1β-induced inflammatory response in human chondrocytes. In addition, the mechanism was also found to inhibit IL-1β-induced inflammation possibly through the regulatory-NF-κB pathway [49].

Cluster III is the relationship between articular cartilage, chondrocytes, metabolism, and inflammation in osteoarthritis. The role of inflammation in osteoarthritis remains unclear. It has been proposed that degradation of articular cartilage is a major factor in the progression of osteoarthritis, and that inflammation and metabolism can modulate each other, thereby regulating cartilage degradation to influence the progression of osteoarthritis. Manoj et al. recently proposed that in the inflammatory response, chondrocytes undergo a metabolic shift regulated by NF-κB activation, which may lead to a shift in cellular metabolism toward glycolysis and lactate dehydrogenase A (LDHA) reprogramming, LDHA could be a new therapeutic target for osteoarthritis [50].

Cluster IV is a number of experiments to observe disease mechanisms associated with osteoarthritis, which are mainly related to changes in lipid peroxidation and related enzymes. ostalowska et al. found a significant increase in the activity of antioxidant-related enzymes in the synovial fluid in patients with knee osteoarthritis (superoxide dismutase, both isoenzymes zinc-copper superoxide dismutase and manganese superoxide dismutase) in patients with knee osteoarthritis [51], leading to a significant decrease in synovial fluid viscosity and further promoting the progression of knee osteoarthritis. An in vitro experiment reveals that chondrocyte lipid peroxidation and collagen oxidation may play a role in the pathogenesis of cartilage aging and osteoarthritis [52].

Cluster V is about the study of signaling pathways related to osteoarthritis. Among the osteoarthritis-related signaling pathways, the most studied pathways are Wnt/β-Catenin Signaling Pathway and NF-κB pathway [53]. Anthriscus sylvestris leaves has a chondroprotective effect on osteoarthritis by inhibiting NF-κB signaling. Aqueous extract of Codium fragile alleviates IL-1β-induced osteoarthritis in rat primary chondrocytes and rat osteoarthritis models via MAPK/NF-κB pathway [54]. Chinese medicine also has an important role in the treatment of osteoarthritis. One study found that Danshensu inhibits IL-1β-induced inflammatory response in chondrocytes and osteoarthritis by inhibiting NF-κB signaling pathway [55]. The remaining related pathways are ERK1/2 pathway, PI3K/AKT Pathway, HOXA1 Pathway etc. [56–58]. These pathways are mainly regulated by microRNAs.

Cluster VI focuses on the expression of cartilage degradation and some related factors and gene pathways in the pathogenesis of osteoarthritis. Deng et al. suggested that YAP/TAZ and NF-κB signaling pathways are mutually antagonistic in inducing matrix degrading enzyme expression and cartilage degradation in the pathogenesis of osteoarthritis [59]. TGFβ may attenuate chondrocyte matrix degradation by enhancing FBXO6-mediated ubiquitination of MMP14 [60].

Cluster VII focuses on the association of osteoarthritis with the above clusters, which have been described separately in the clusters above.

Cluster VIII is the role of activation of some proteins, pathophysiological processes, and bioregulatory factors in the development of osteoarthritis. Angiogenesis has an important role in the development of osteoarthritis. In rheumatoid arthritis, circulating leukocytes migrate excessively into the inflamed joint and need to form new blood vessels to provide nutrients and oxygen to the hypertrophied joint, and pro-angiogenic factors play an important role in this. GATA4 regulates angiogenesis and the persistence of inflammation in rheumatoid arthritis, according to a study [61]. WTD attenuates rheumatoid arthritis by inhibiting angiogenesis and regulating the PI3K/AKT/mTOR/HIF-1α pathway [62]. A large body of research evidence suggests that inhibition of angiogenesis may be an effective therapeutic idea for the treatment of osteoarthritis.

Cluster IX focuses on the relationship between the NF-κB pathway and rheumatoid arthritis. the relationship between the NF-κB pathway and the development of rheumatoid osteoarthritis is relatively well studied. This is mainly due to the fact that the NF-κB pathway is closely associated with the progression of inflammation. Recently, it has been found that KP-10/Gpr54 attenuates rheumatoid arthritis by inactivating NF-κB and MAPK signaling in macrophages [63]. It has also been proposed that EtOAc extract of H. attenuatum Choisy suppresses inflammation by inhibiting NF-κB and MAPK pathways and regulating intestinal microbiota [64].

## Conclusions

Bibliometric analysis shows that the research progress of oxidative stress and osteoarthritis is rapid. China is a major producer, and the United States has made many outstanding breakthroughs in this field. OSTEOARTHRITIS AND CARTILAGE reported the latest research and Cartilage developments in the field. In recent years, the role of nf-kappa b molecular pathways in this field has become a focus of research.

## Supplementary Information

**Additional file 1. **

## Data Availability

All data generated during this study are included in this published article and its supplementary information files. The analysis during the study can be obtained from the corresponding author Qing Zhang on reasonable request.
